# Exploring the link between walking and lung cancer risk: a two-stage Mendelian randomization analysis

**DOI:** 10.1186/s12890-024-02906-0

**Published:** 2024-03-13

**Authors:** Fangjun Chen, Chutong Lin, Xing Gu, Yingze Ning, Huayu He, Guangliang Qiang

**Affiliations:** 1 Department of Thoracic Surgery, China-Japan Friendship Institute of Clinical Medicine, No.2 Yinghua East Street, Chaoyang District, Beijing, 100029 China; 2 Department of Thoracic Surgery, Peking University Third Hospital, Haidian District, No.49 Huayuan North Road, Beijing, 100191 China; 3 College of Foreign Languages, Chongqing Medical University, Yuzhong District, No.1 Yixueyuan Road, Chongqing, 400016 China

**Keywords:** Lung cancer, Walk, Mendelian randomization, Sedentary behaviors

## Abstract

**Background:**

Previous observational research showed a potential link between physical activities such as walking and the risk of lung cancer. However, Mendelian randomization (MR) studies suggested there was no association between moderate to vigorous physical activity and lung cancer risk. We speculated that specific physical activities may be associated with lung cancer risk. Consequently, we conducted an MR study to examine the potential relationship between walking and the risk of lung cancer.

**Methods:**

We collected genetic summary data from UK Biobank. After excluding SNPs with F values less than 10 and those associated with confounding factors, we conducted a MR analysis to assess the causal effects between different types of walk and lung cancer. We also performed sensitivity analysis to validate the robustness of our findings. Finally, we analyzed the possible mediators.

**Results:**

MR analysis showed number of days/week walked for 10 + minutes was associated with a reduced risk of lung cancer risk (OR = 0.993, 95% CI = 0.987–0.998, *P* = 0.009). Additionally, usual walking pace was identified as a potentially significant factor in lowering the risk (OR = 0.989, 95% CI = 0.980–0.998, P = 0.015). However, duration of walks alone did not show a significant association with lung cancer risk (OR = 0.991, 95%CI = 0.977–1.005, *P* = 0.216). The sensitivity analysis confirmed the robustness of these findings. And number of days/week walked for 10 + minutes could affect fed-up feelings and then lung cancer risk. There was a bidirectional relationship between usual walking pace and sedentary behaviors (time spent watching TV).

**Conclusion:**

The study unveiled a genetically predicted causal relationship between number of days/week walked for 10 + minutes, usual walking pace, and the risk of lung cancer. The exploration of potential mediators of walking phenotypes and their impact on lung cancer risk suggests that specific physical activities may reduce the risk of lung cancer.

**Supplementary Information:**

The online version contains supplementary material available at 10.1186/s12890-024-02906-0.

## Introduction

Lung cancer, a prevailing malignancy, is the leading cause of global cancer-related mortality [1]. Given the substantial healthcare and economic implications associated with lung cancer, it becomes imperative to discern modifiable and preventable factors that could contribute to primary prevention. This approach can yield substantial reductions in cancer risk by reducing exposure to hazards and altering behaviors. The potential risk factors of lung cancer include smoking, pulmonary fibrosis, genetic history, diets, air pollution, and insufficient physical activity [2–4].

Previous observational studies have unveiled an inverse correlation between physical activities, including walking, and the risk of lung cancer [5–7]. However, substantial studies confined their findings to current or past smokers. And recent MR analyses suggest there was no association between moderate to vigorous and lung cancer risk [8, 9]. Therefore, it is imperative to elucidate the connection between walking and the risk of lung cancer with a robust study. This is critical for comprehending potential risk factors associated with lung cancer, patient education, and patient anxiety alleviation.

The inherent limitations of conventional design prevent the available observational studies from fully eliminating the reverse causality and confounding factors, which may lead to association and conclusion biases [10]. The randomized controlled trials (RCTs) is often ethically and practically infeasible due to the personnel costs and the time-consuming follow-ups [11].

MR is a technique using genetic variations as instrumental variables (IVs) and can discover causality in cases of unobserved confounding factors and reverse causality. MR could mitigate the impact of environmental confounding factors since alleles are randomly assigned at conception, and diseases cannot affect genotype. This is helpful to reduce reverse causality [12, 13].

Therefore, in this study, we conducted a two-sample summary data MR analysis to evaluate the association between walking and lung cancer risk, and then analyzed the possible mediators.

## Methods

As the data included in this study are publicly available, we did not apply for any specific ethical consent or review from any participants of the GWAS above.

In this study, we performed a MR analysis to examine the causal effect of walking on lung cancer using GWAS summary statistics. This IVs analysis simulated RCTs with the random assignment of SNPs in offspring (unrelated to confounders such as gender and age). This MR design must fulfill three assumptions: (i) genetic instruments predict the exposure of interest (*P* < 5 × 10–8); (ii) genetic instruments are independent of potential confounding factors; and (iii) genetic instruments affect the outcome through risk factors only [14].

We collected the summary data of walk from the IEU OpenGWAS project. The summary data for walk-related phenotypes were obtained from UK Biobank (Neale Lab), in which the data for number of days/week walked for 10 + minutes, duration of walks and usual walking pace included 331,654, 288,266 and 335,349 subjects, respectively. The data for lung cancer were obtained from UK Biobank, including 374,687 subjects (2671 diagnosed with lung cancer and 372,016 without lung cancer). The data for fed-up feeling were obtained from UK Biobank(MRC-IEU), including 453,071 subjects. The data for time spent watching TV were collected from the UK Biobank(MRC-IEU), including 437,887 subjects. All subjects were of European descent. Details are shown in Table 1.

**Table 1:** Details of exposures and outcome included in MR analyses

GWAS ID	Source	Phenotype	Participants
ukb-a-506	UK Biobank (Neale Lab)	Number of days/week walked 10 + minutes	331,654
ukb-a-507	UK Biobank (Neale Lab)	Duration of walks	288,266
ukb-a-513	UK Biobank (Neale Lab)	Usual walking pace	335,349
ukb-b-19809	UK Biobank (MRC-IEU)	Fed-up feelings	453,071
ukb-b-5192	UK Biobank (MRC-IEU)	Time spent watching TV	437,887
ieu-b-4954	UK Biobank	Lung cancer	374,687

The IVs of the target phenotypes were identified according to the following criteria proposed by Martin Bahls et al. (1) SNPs at genome-wide significance (*P* < 5 × 10–8); (2) SNP clustering using the PLINK algorithm (LD r2 < 0.001, distance kb > 10 mB); and (3) removal of SNPs that exhibit potential pleiotropic effects [15]. In addition, we collected SNPs of target phenotypes in the PhenoScanner database (http://www.phenoscanner.medschl.cam.ac.uk/) when analyzing the effect of target phenotypes on lung cancer risk, so as to assess whether IV was associated with lung cancer confounders. We also excluded SNPs associated with confounding factors, such as smoking, alcohol, family history of lung cancer, air pollution (including PM2.5, PM2.5–10, PM10, nitrogen dioxide and nitrogen oxides), idiopathic pulmonary fibrosis (IPF), chronic obstructive pulmonary disease (COPD) and occupational exposure (dust,asbestos fibers, coke oven emissions, crystalline silica) [16–21]. The correlation strength between SNPs and target phenotypes was expressed as an F-statistic [22]. In general, an F-statistic > 10 indicates a strong correlation between IVs and target phenotypes.

### MR analysis

To address the potential pleiotropic effects of genetic variants, we used 3 MR methods in this study to assess the causal effects of the exposure of interest on the target outcome. The inverse variance weighted (IVW) approach was used as the principal analysis, which combined the Wald ratio of each SNP to the outcome and gained summary causal estimates. This approach allows for over-dispersion. In addition, other MR methods supplementary to the IVW, such as MR-Egger regression and weighted median, can provide more reliable estimates in a wider range of situations. MR-Egger regression can provide tests for unbalanced pleiotropy and considerable heterogeneity, while it requires a larger sample size for same under-exposure variation [22]. The weighted median approach offers consistent effect estimates when the weighted variance provided by the horizontal pleiotropy is at least half valid [23].

### Sensitivity analysis

Horizontal pleiotropy will occur when genetic variation associated with the exposure of interest directly affect the outcome through multiple channels other than the hypothesized exposure. Therefore, we further performed Cochran’s Q test, funnel plot, leave-one-out analysis, MR-PRESSO and MR-Egger intercept test to detect the pleiotropy and to assess the robustness of the results. Specifically, heterogeneity would be identified if the *p*-value of the Cochran Q test was less than 0.05, at which point a random effects model was required. The horizontal pleiotropy would be detected if the *p*-value of the MR-Egger intercept test and MR-PRESSO were less than 0.05. We also performed a leave-one-out analysis by which each exposure-related SNP was discarded in turn to repeat the IVW analysis, so as to determine whether causal estimates were driven by any single SNP.

The Bonferroni-adjusted *p*-values below 0.01 (0.05/5 exposures) were considered significant and showed a strong association when lung cancer was taken as an outcome. And *p* values of 0.01–0.05 was considered potentially significant.

All analyses were performed through the packages TwoSampleMR (version 0.5.6), gwasglue (version 0.0.0.9000) and VariantAnnotation (version 1.44.1) in R (version 4.2.2).

## Results

### Selected genetic variants for exposure and outcome

In our analysis of the relationship between these factors and lung cancer risk, we finally extracted 7, 3, 23, 13 and 100 SNPs associated with number of days/week walked for 10 + minutes, duration of walks, usual walking pace, fed-up feelings, and time spent watching TV as IVs, respectively. This selection was made following the exclusion of SNPs with F statistic below 10 and those associated with lung cancer's confounding factors (smoking, alcohol, family history of lung cancer, air pollution, IPF, COPD and occupational exposure). When analyzing the relationship between number of days/week walked for 10 + minutes, usual walking pace, fed-up feelings and lung cancer, we excluded rs3004179, rs13107325, rs1652376, rs34898535, rs7124682, rs7896518, rs9972653, and rs4630591, which were all associated with alcohol consumption. When analyzing the relationship between time spent watching TV and lung cancer, we excluded SNPs associated with smoking (rs71658797 and rs75499503) and alcohol (rs11714337, rs2352984, rs4339469, rs62199883, and rs872169). However, we did not find SNPs associated with other confounders.

The corresponding F-statistic ranges were 116–158, 30–38, 11–20, 10–16 and 16–46 respectively. These values exceeded the standard cutoff values (> 10), indicating a robust instrumental strength. In the analysis of the relationship between number of days/week walked for 10 + minutes and fed-up feelings, we extracted 6 relevant SNPs as IVs. Similarly, when analyzing the association between usual walking pace and time spent watching TV, we extracted 21 relevant SNPs as IVs. The corresponding F-statistic values were all higher than the cutoff values (> 10).

Details on the variants used to construct the above phenotypes’ IVs, are shown in Supplementary File 1.

### MR analysis

**Fig. 1:**
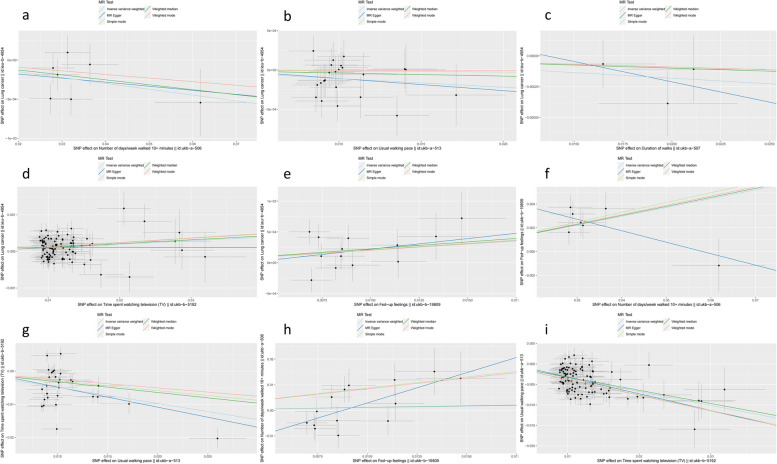
Scatter plot. **a** The effect of number of days/week walked 10 + minutes on lung cancer. **b** The effect of usual walking pace on lung cancer. **c** The effect of duration of walks on lung cancer. **d** The effect of time spent watching TV on lung cancer. **e** The effect of fed-up feelings on lung cancer. **f** The effect of number of days/week walked 10 + minutes on fed-up feelings. **g** The effect of usual walking pace on time spent watching TV. **h** The effect of fed-up feelings on number of days/week walked 10 + minutes. **i** The effect of time spent watching TV on usual walking pace

The IVW results showed that number of days/week walked for 10 + minutes (OR = 0.993, 95% CI = 0.987–0.998, *P* = 0.009) and time spent watching TV (OR = 1.009, 95% CI = 1.005–1.014, *P* = 2.9e-5) affected the risk of lung cancer. Usual walking pace (OR = 0.989, 95% CI = 0.980–0.998, *P* = 0.015) and fed-up feelings (OR = 1.021, 95% CI = 1.005–1.037, *P* = 1.3e-7) were potentially significant in relation to lung cancer risk. In addition, number of days/week walked for 10 + minutes was significantly associated with fed-up feelings (OR = 1.095, 95% CI = 1.063–1.128, *P* = 2.5e-9), but fed-up feelings did not affect number of days/week walked for 10 + minutes (OR = 1.116, 95% CI = 0.722–1.725, *P* = 0.621). Usual walking pace was significantly associated with time spent watching TV (OR = 0.821, 95% CI = 0.771–0.875, *P* = 8.9e-10), and time spent watching TV may affect usual walking pace (OR = 0.714, 95% CI = 0.685–0.744, *P* = 3.4e-58). Details are shown in Table 2 and Fig. 1.

**Table 2:** Mendelian randomization

		IVW method		MR-Egger		Weighted median	
Exposure	Outcome	OR (95% CI)	*P*-value	OR (95% CI)	*P*-value	OR (95% CI)	*P*-value
Number of days/week walked 10 + minutes	Lung cancer	0.993 (0.987–0.998)	0.009	0.995 (0.966–1.024)	0.742	0.994 (0.986–1.001)	0.094
Duration of walks	Lung cancer	0.991 (0.977–1.005)	0.216	0.968 (0.848–1.104)	0.710	0.995 (0.978–1.012)	0.566
Usual walking pace	Lung cancer	0.989 (0.980–0.998)	0.015	0.985 (0.932–1.041)	0.523	0.996 (0.984–1.008)	0.523
Time spent watching TV	Lung cancer	1.009 (1.005–1.014)	2.9e-5	0.996 (0.975–1.017)	0.710	1.011 (1.005–1.016)	1.8e-4
Time spent watching TV	Usual walking pace	0.714 (0.685–0.744)	3.4e-58	0.667 (0.554–0.804)	4.5e-5	0.725 (0.691–0.761)	2.7e-39
Fed-up feelings	Lung cancer	1.021 (1.005–1.037)	0.012	1.035 (0.955–1.122)	0.418	1.023 (1.002–1.044)	0.028
Fed-up feelings	Number of days/week walked 10 + minutes	1.116 (0.722–1.725)	0.621	1.123 (0.684–1.844)	0.646	10.63 (1.720–65.75)	0.026
Number of days/week walked 10 + minutes	Fed-up feelings	1.095 (1.063–1.128)	2.5e-9	1.088 (0.754–1.571)	0.680	1.087 (1.046–1.131)	2.6e-5
Usual walking pace	Time spent watching TV	0.821 (0.771–0.875)	8.9e-10	0.712 (0.547–0.928)	2.0e-2	0.826 (0.770–0.888)	1.7e-7

### Two-step MR analysis

The total effect of the exposures to the outcome was β1, the effect of the exposures to the mediators was β2, and the effect of the mediators to the outcome was β3. Then the total effect of the exposures to the outcome was β1, the mediating effect was β2*β3, the direct effect was β1-β2*β3, and the share of the mediating effect share was β2*β3/β1.

**Fig. 2:**
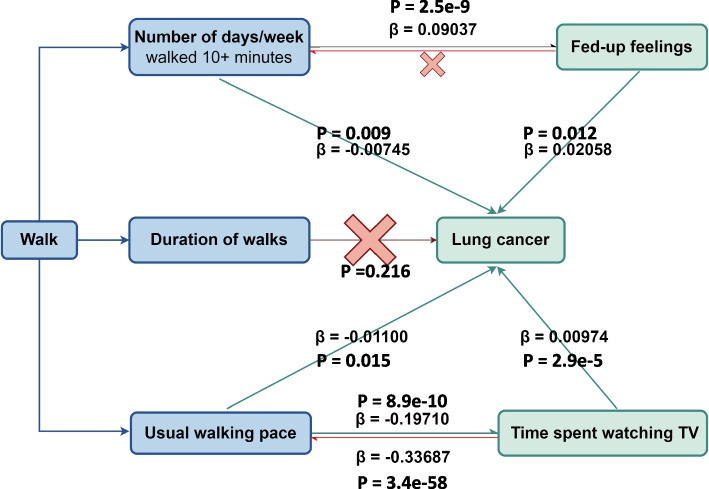
Two-step MR analysis

When analyzing the effect of number of days/week walked for 10 + minutes on the risk of lung cancer, the mediating effects of fed-up feelings was 0.00186, accounting for 25.0%. But fed-up feelings could not affect number of days/week walked 10 + minutes. When analyzing the effect of usual walking pace on the risk of lung cancer, the mediating effect of time spent watching TV was 0.00192, accounting for 17.5%. Interestingly, time spent watching TV may affect usual walking pace and then lung cancer risk, in which the mediating effect of usual walking pace was 0.0037, accounting for 38.0%. When analyzing the impact of number of days/week walked for 10 + minutes, fed-up feelings, usual walking pace, and the time spent watching TV on lung cancer risk, there was no overlap of SNPs between IVs associated with the direct factors and those associated with the mediating factors. Details are shown in Fig. 2**.** In addition, all data in this study were obtained from UK Biobank. In order to assess the reliability of the study, we explored the impact of sample overlapping rate on the conclusions in Bias and Type 1 error rate for Mendelian randomization with sample overlap (https://sb452.shinyapps.io/overlap/). However, previous studies haven’t showed the possible OR between exposures and outcomes, so we hypothesized the OR of possible protective factors was 0.3 or 0.9, and the OR of possible risk factors was 1.1 or 3.0 when lung cancer is the outcome. The same method was adopted when the outcome is other factors. The results suggested that, even though all data in this study are from UK Biobank, the sample overlapping rate may not affect the reliability of the conclusions, especially when compared with the OR obtained in this study. Moreover, when analyzing the relationship between number of days/week walked for 10 + minutes and fed-up feelings, the sample overlapping rate may also not affect the final conclusion. However, when analyzing the relationship between usual walking pace and time spent watching TV, the conclusion may be greatly affected by the sample overlapping rate, possibly because the sample overlapping rate and time spent watching TV are categorical ordinal data, rather than binary or continuous variables. The results are shown in Table 3.

**Table 3:** Probability of Type I error when sample overlapping rate is 100%

Exposure	Outcome	OR = 0.9 or 1.1	OR = 0.3 or 3.0
Number of days/week walked 10 + minutes	Lung cancer	0.05	0.05
Usual walking pace	Lung cancer	0.05	0.05
Duration of walks	Lung cancer	0.05	0.05
Time spent watching TV	Lung cancer	0.05	0.08
Time spent watching TV	Usual walking pace	0.09	0.41
Fed-up feelings	Lung cancer	0.05	0.06
Fed-up feelings	Number of days/week walked 10 + minutes	0.18	0.82
Number of days/week walked 10 + minutes	Fed-up feelings	0.05	0.07
Usual walking pace	Time spent watching TV	0.08	0.37

### Sensitivity analysis

**Fig. 3:**
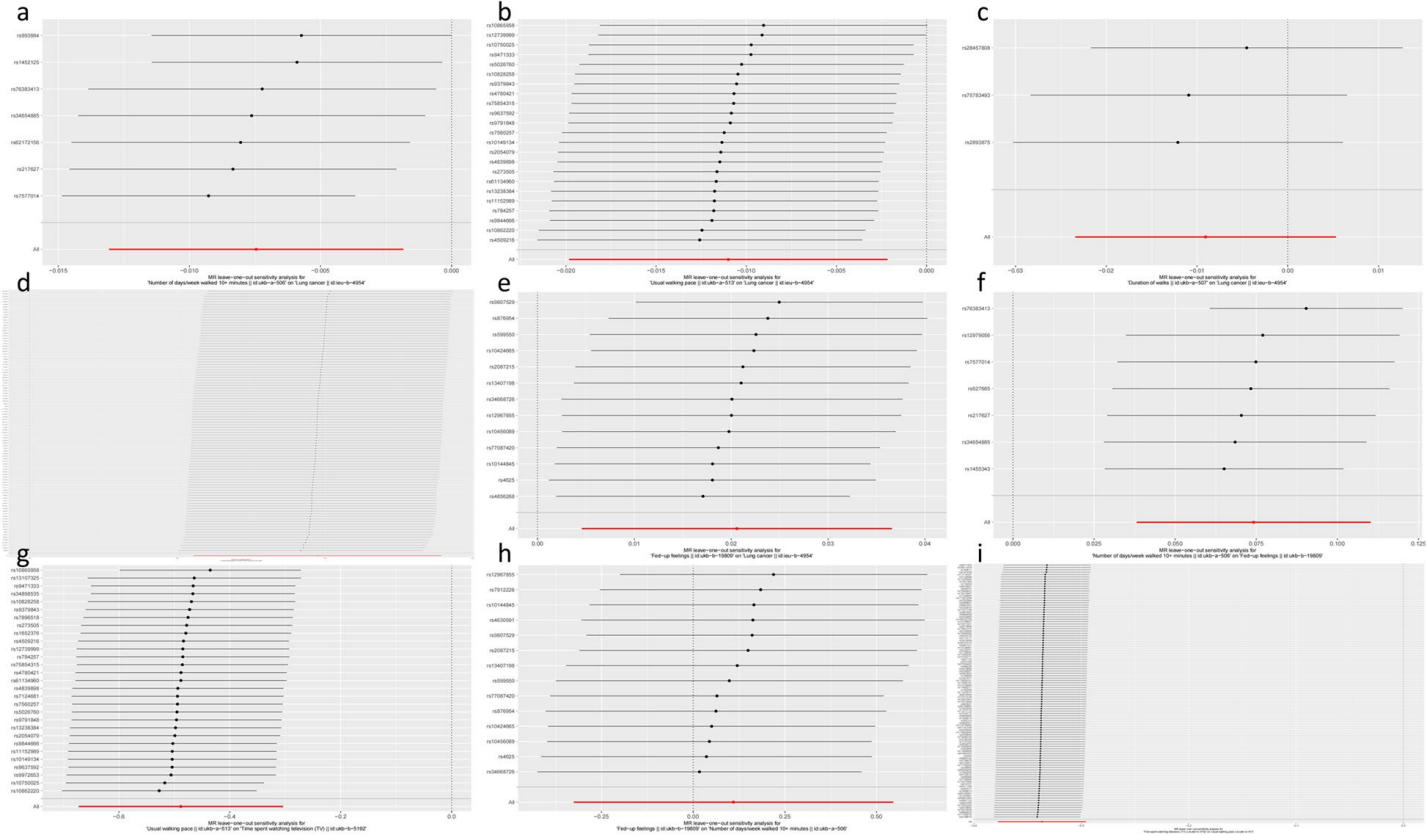
Leave-one-out analysis. **a** The effect of number of days/week walked 10 + minutes on lung cancer. **b** The effect of usual walking pace on lung cancer. **c** The effect of duration of walks on lung cancer. **d** The effect of time spent watching TV on lung cancer. **e** The effect of fed-up feelings on lung cancer. **f** The effect of number of days/week walked 10 + minutes on fed-up feelings. **g** The effect of usual walking pace on time spent watching TV. **h** The effect of fed-up feelings on number of days/week walked 10 + minutes. **i** The effect of time spent watching TV on usual walking pace

**Fig. 4:**
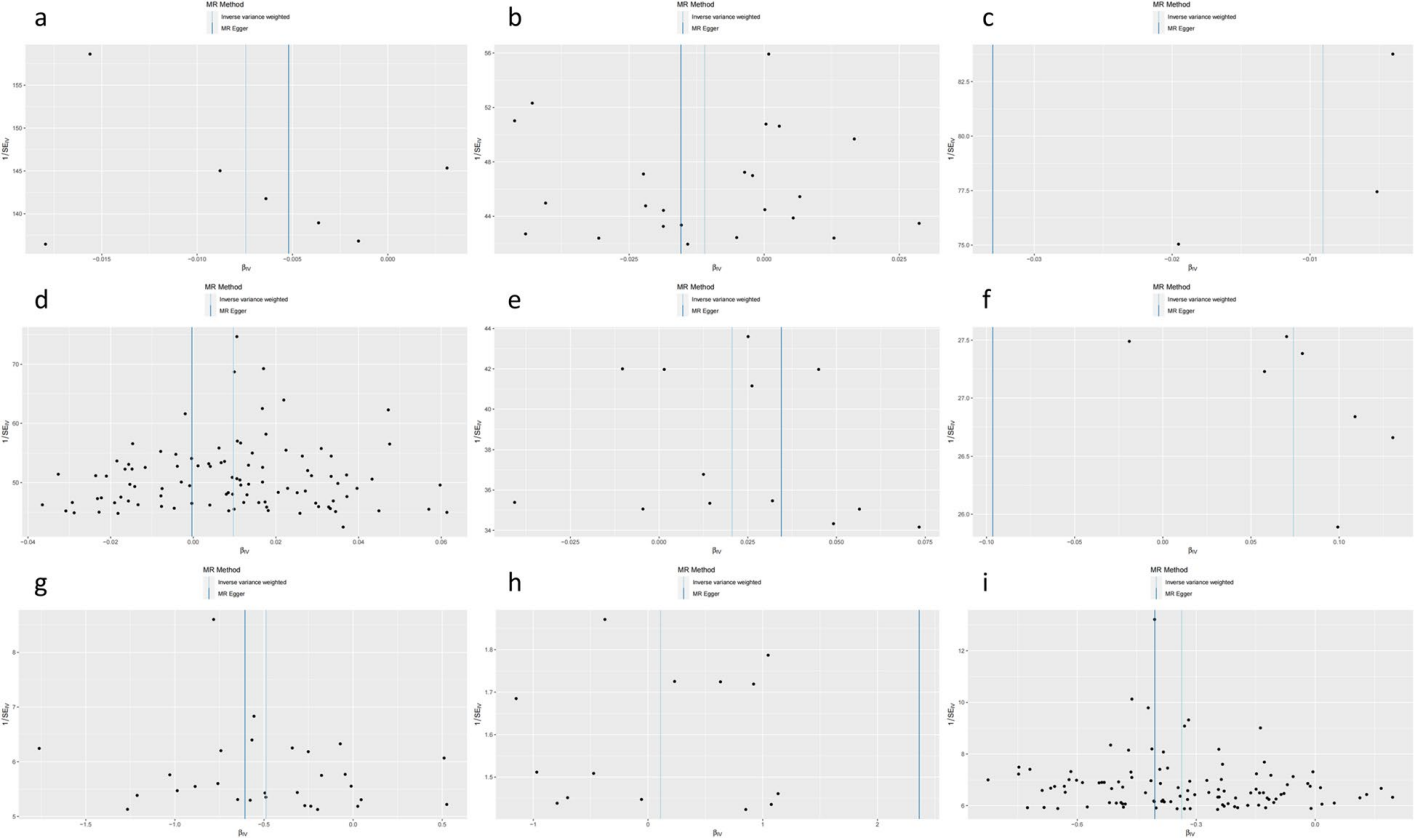
Funnel plot. **a** The effect of number of days/week walked 10 + minutes on lung cancer. **b** The effect of usual walking pace on lung cancer. **c** The effect of duration of walks on lung cancer. **d** The effect of time spent watching TV on lung cancer. **e** The effect of fed-up feelings on lung cancer. **f** The effect of number of days/week walked 10 + minutes on fed-up feelings. **g** The effect of usual walking pace on time spent watching TV. **h** The effect of fed-up feelings on number of days/week walked 10 + minutes. **i** The effect of time spent watching TV on usual walking pace

A series of sensitivity analyses were performed to assess the robustness of the above results, including Cochran’s Q test, MR-Egger intercept test, leave-one-out analysis, MR-PRESSO and funnel plot, among them, MR-Egger intercept test had *p*-values > 0.05 and did not detect pleiotropy. However, when analyzing the relationship between usual walking pace and time spent watching TV, the *P*-value of MR-PRESSO was still less than 0.05 after removing the SNP with pleiotropy. Some of Cochran’s Q-tests showed heterogeneity, for which the data were restricted to a random effects model only (Table 4). The leave-one-out analysis showed no SNP-driven results (Fig. 3), and the funnel plot was symmetric (Fig. 4).

**Table 4:** Sensitivity analysis

Exposure	Outcome	MR-Egger intercept	Cochran’s Q test	MR-PRESSO
Number of days/week walked 10 + minutes	Lung cancer	0.884	0.314	0.363
Usual walking pace	Lung cancer	0.876	0.573	0.568
Duration of walks	Lung cancer	0.781	0.641	-
Time spent watching TV	Lung cancer	0.323	0.088	0.094
Time spent watching TV	Usual walking pace	4.1e-12	0.466	-
Fed-up feelings	Lung cancer	0.736	0.239	0.254
Fed-up feelings	Number of days/week walked 10 + minutes	0.029	0.045	0.054
Number of days/week walked 10 + minutes	Fed-up feelings	0.976	0.755	0.822
Usual walking pace	Time spent watching TV	0.292	0.007	0.013

## Discussion

Lung cancer is characterized by high global incidence and mortality, and previous studies have not elucidated the precise nature and direction of the association between walking and lung cancer risk. Therefore, in this study, we conducted several MR methods to evaluate the causality between three walking phenotypes and the risk of lung cancer by using data from UK Biobank. The results showed that number of days/week walked for 10 + minutes can lower the risk of lung cancer. Additionally, usual walking pace emerged as a potentially significant factor, which provides new insights into their genetic-level association [24–27].

In addition, we demonstrated that lung cancer risk was associated with fed-up feelings and the time spent watching TV. Number of days/week walked for 10 + minutes can affect fed-up feelings, while usual walking pace can affect time spent watching TV. This showed a potential possibility that number of days/week walked for 10 + minutes and usual walking pace may affect the risk of lung cancer. We also observed a positive correlation between number of days/week walked for 10 + minutes and fed-up feelings.

Notably, previous MR studies have shown that lung cancer risk is not associated with depression and moderate to vigorous physical activity [9, 28]. However, numerous observational studies have suggested a possible association, so we focused on specific physical activities (walking) and specific emotion (fed-up feelings), and examined their relationship with lung cancer risk through MR study. Television watching is considered a leisure sedentary behavior, and previous MR Study have suggested that it may influence lung cancer risk through the amount of smoking [29].

Previous studies have shown that walking can affect the emotions of subjects, especially the elderly and women [30, 31], which is initially consistent with the findings of the present study that walking can affect fed-up feelings. However, there are no studies showing the relationship between specific types of walking, such as frequency, duration, and speed, and lung cancer, or between specific emotions (fed-up feelings) and lung cancer. In addition, although some studies have shown that sedentary behaviors can affect smoking and then lung cancer risk, there are no studies suggesting a possible association between walking pace and sedentary behaviors. More importantly, the subjects included in the study are from UK Biobank, and the possible bidirectional causality between usual walking pace and time spent watching TV causality is largely caused by the sample overlapping rate, but it seems not to affect the stability of the present conclusions.

In general, the IVW method exhibits notably higher statistical power than other MR techniques, particularly the MR-Egger [32]. The MR-Egger results with low statistical power have broader confidence and insignificant *p*-values, compared with IVW in this study [11]. Therefore, IVW is commonly employed as the primary method for identifying potentially important results in MR analyses. When analyzing the effect of time spent watching TV on the risk of lung cancer, although the direction of MR-Egger's β-value was opposite to that of IVW and Weighted median, MR-Egger did not showed significant results (*P* > 0.05). And the present study mainly referred to the results of IVW, so we concluded that time spent watching TV may affect lung cancer risk. In addition, sensitivity analyses and additional MR methods were used to ensure the robustness of IVW estimates.

If there was horizontal pleiotropy, IVW estimates may be biased. In this case, MR-Egger estimates should be considered as a reference, as MR-Egger can adjust the IVW analysis by allowing the horizontal pleiotropy effects of all SNPs to be unbalanced or oriented [32–34].

However, the study has several limitations. Firstly, the study participants are of European descent, which may not be directly applicable to other ethnic groups with different lifestyles and cultural backgrounds. Secondly, MR analyzes are reliant on causal hypotheses thorough random assignment of genetic variants. Consequently, it is difficult to distinguish mediation from pleiotropy using MR methods. Finally, although some studies have shown that walking and emotions are associated with lung cancer risk, they did not analyze the relationship between specific types of walking or emotions and lung cancer risk. Therefore, this study is only a preliminary exploration, and further research is needed to confirm the findings.

In conclusion, using large-scale genetic summary data, this study primarily elucidated the evidence for a causal relationship between physical activity (number of days/week walked for 10 + minutes) and lung cancer, and analyzed the underlying mechanism. Given the global incidence and mortality of lung cancer and previous MR studies showing that moderate to vigorous exercise does not reduce the risk of lung cancer [7, 35–37], much attention should be paid to the positive impact of specific physical activities, particularly exercise frequency, thus reducing the incidence of lung cancer.

### Supplementary Information

**Supplementary Material 1.**

## Data Availability

The data in the study can be obtained from the corresponding author upon reasonable request. This study was conducted using the IEU OpenGWAS project. It is an open access resource and researchers can use it by registering at https://gwas.mrcieu.ac.uk/.

## Abbreviations

CI: Confidence interval
COPD: Chronic obstructive pulmonary disease
GWAS: Genome-wide association studies
IPF: Idiopathic pulmonary fibrosis
IVs: Instrumental variables
IVW: Inverse variance weighted
MR: Mendelian Randomization
OR: Odds ratio
RCTs: Randomized controlled trials
SNPs: Single nucleotide polymorphisms
